# Mr. Lawson Tait on Lumbar Colotomy

**Published:** 1898-07-09

**Authors:** 


### MR. LAWSON TAIT ON LUMBAR COLOTOMY.

Mr. Lawson Tait, after his own manner, tilts at the text-books, and especially at the teachings of anatomical surgeons. He also disapproves of inguinal colotomy, and pins his faith on lumbar colotomy done after a method which he describes. It is difficult, however, for a critic to avoid the observation that after all his onslaughts on the anatomists, and all his assertions as to the variability of human anatomy, he has to come back to anatomy for his guide-post. After describing how he places the patient on her right side with her knees bent up to steady her, he says that he places a pillow "under her right loin, so as to put on the stretch that which is a guide to the whole thing—a strip of tendon which marks the union of the fascias encasing the external oblique muscle, the internal oblique, the transversalis on the one side, and the lattissimus dorsi (or its tendon) and the quadratus lumborum on the other." After dividing the skin over this tight band and absolutely in its direction the surgeon has only to open the glistening fascia in the same position, and observe the direction of the muscular fibres, which he must then separate by means of his forefingers. In doing this he must bear only one route in mind, and that is as nearly as possible to the umbilicus. The direction in which the muscular fibres will be found to separate will at first be parallel to the axis of the patient's trunk, and afterwards at right angles to that axis; but there is no need to trouble to identify the muscles. Then he will come upon the transversalis fascia, which he must notch, and on getting his forefingers through it he must search towards the body of the vertebra till he finds the base of the kidney. Having found this, he must remember that in front of the kidney, and thus in front of his finger, but separated from it by some loose fat, lies the descending colon. The surgeon will then "take two or three pairs of catch forceps and pull layer after layer of fat boldly and freely up into the wound, leaving the forceps on each layer as he lands it outside, and proceed in this way till he sees the unmistakable gut. If he keeps the points of his forceps well downwards and pointing well forwards towards the umbilicus, he cannot miss the gut. When the gut is missed it is always by dissecting-room people, who go over it instead of at it. Having found the gut haul it freely and fearlessly up out of the wound, cut it nearly through, and stitch with an all-round suture into the *lower* angle of the wound, carefully replacing all fringes of fat," or removing them by the fingers if they have been much injured by the forceps. The rest of the wound is then to be closed. The result of this operation is that the whole of the gut has been pulled through the crosswise made opening in the muscles, so that the almost constant tension involved in the all-active positions of the body secures the closure of the aperture of the gut. Mr. Lawson Tait goes on to say that in a badly-done colotomy only a bit of the circumference of the gut is pulled up to or out of the muscular aperture, the bulk of it being left as before inside, and the gut is emptied through the relief passage only by regurgitation from below, and generally when the whole of it is distended by hardened fæces, whereas after colotomy done as he recommends it should be done all the motion comes through the wound at once, and after some practice there comes marvellous control over the passage. This ought to be a good operation. The pulling out of the entire circumference of the bowel is certainly good, and is, indeed, one of the main factors in the success of the inguinal colotomy. But, after all, we cannot avoid the observation that the described method of what we may call "fishing for gut" shows considerable confidence in the correctness of the observations of the anatomists, notwithstanding all that is said about them.

